# A transcription factor PU.1 is critical for *Ccl22* gene expression in dendritic cells and macrophages

**DOI:** 10.1038/s41598-018-37894-9

**Published:** 2019-02-04

**Authors:** Takuya Yashiro, Shiori Nakano, Kurumi Nomura, Yuna Uchida, Kazumi Kasakura, Chiharu Nishiyama

**Affiliations:** Department of Biological Science and Technology, Faculty of Industrial Science and Technology, Tokyo University of Science, 6-3-1 Niijuku, Katsushika-ku, Tokyo 125-8585 Japan

## Abstract

The chemokine CCL22 is predominantly produced by dendritic cells (DCs) and macrophages. CCL22 acts on CCR4-expressing cells including Th2 and Treg. Although a correlation between the CCL22-CCR4 axis and allergic diseases has been established, the mechanism of monocyte lineage-specific *Ccl22* gene expression is largely unknown. In the current study, we investigated transcriptional regulation of the *Ccl22* gene in DCs and macrophages. Using reporter assays, we identified the critical *cis*-enhancing elements at 21/−18 and −10/−4 in the *Ccl22* promoter. Electrophoretic mobility shift assays proved that transcription factor PU.1 directly binds to the *cis*-elements. Knockdown of PU.1 markedly decreased *Ccl22* expression in bone marrow-derived DCs (BMDCs) and BM macrophages (BMDMs). Chromatin immunoprecipitation assays revealed that PU.1 bound to the *Ccl22* promoter in not only BMDCs and BMDMs, but also splenic DCs and peritoneal macrophages. LPS stimulation increased the amount of PU.1 recruited to the promoter, accompanied by upregulation of the *Ccl22* mRNA level, which was diminished by *Spi1* knockdown. We identified similar *cis*-elements on the human *CCL22* promoter, which were bound with PU.1 in human monocytes. Taken together, these findings indicate that PU.1 transactivates the *Ccl22* gene in DCs and macrophages by directly binding to the two elements in the promoter.

## Introduction

Chemokines are chemo-attractant proteins which regulate the migration of different subsets of leukocytes during immune responses. CCL22 (also called macrophage-derived chemokine, MDC) is one of the chemokines mainly produced by myeloid cells such as macrophages and DCs in the steady state^1^. CCL22 and a closely related chemokine, CCL17, tandemly locate on human chromosome 16 and mouse chromosome 8. These chemokines share the same receptor, CCR4, predominantly expressed by Th2 cells. Therefore, these chemokines are considered to be key mediators in the development of Th2-dominant diseases such as atopic dermatitis (AD) and asthma. Indeed, serum levels of CCL22 and CCL17 are significantly elevated in AD patients^2^. It has been reported that CCL22 and CCR4 are also expressed in several types of tumor cells and Foxp3^+^ Treg, respectively. This CCL22-CCR4 axis is thought to attract Treg into the tumor microenvironment to evade the immune attack^3–5^. A CCR4 antagonist is useful for alleviating immune suppression and preventing tumor growth^6,7^.

PU.1 is a transcription factor specifically expressed in hematopoietic lineages. It is well known that expression level of PU.1 determines the cell fate during development of B cells, macrophages, and granulocytes from hematopoietic stem cells^8–10^. A study using PU.1 reporter mice demonstrated that PU.1 is highly expressed in myeloid DCs, in contrast to a low level expression of PU.1 in plasmacytoid DCs^11^. PU.1 binds to Ets motifs as a monomer and binds to EICE motifs as a heterodimer PU.1/IRF4 or PU.1/IRF8. C/EBPα and β, and c-Jun are also known to form a complex with PU.1^12^. We have demonstrated that PU.1 regulates the expression of DC-characteristic genes including *Cd80*, *Cd86*, *Ciita*, *Tnf-α*, and *Tnfsf4*^13–16^. Moreover, previous studies have shown that PU.1 is involved in macrophage-specific gene expression^17–19^.

Despite its critical involvement in several allergic diseases, the expression mechanism of the *Ccl22* gene is not fully understood. In the current study, we found that PU.1 transactivates the *Ccl22* gene both in DCs and macrophages via the two Ets motifs in the promoter.

## Results

### Ccl22 promoter activity in DC- and Macrophage-cell lines

**Figure 1:**
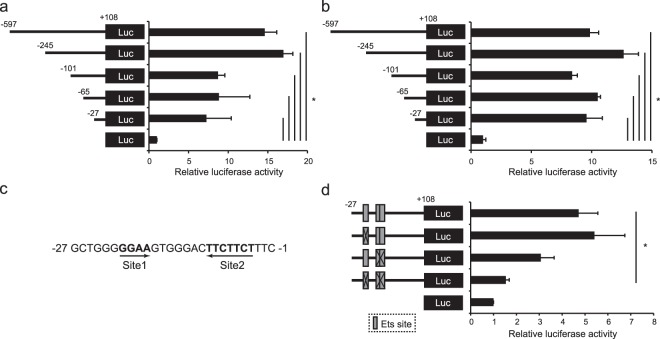
Analysis of *cis*-element in the *Ccl22* promoter. JAWSII cells (**a**) and RAW264.7 cells (**b**) were transfected with reporter plasmids for renilla luciferase as an internal control and with reporter plasmids for firefly luciferase containing various lengths of the mouse *Ccl22* promoter. (**c**) Sequences of the −27/−1 region of the mouse *Ccl22* promoter. Two Ets motifs, designated Site1 and Site 2, are indicated in bold. (**d**) JAWSII cells were transfected with reporter plasmids of WT or mutant promoters lacking the Ets motif(s) at the indicated sites. All results are shown as means + S.D.s (*n* = 3). Similar results were obtained in three independent experiments. *p < 0.05.

To determine the region critical for promoter activity, we generated a series of reporter vectors, and carried out luciferase assays by transfection of monocyte cell lines with the reporter plasmids. A luciferase assay using JAWSII, a mouse DC cell line, as the host showed that the luciferase activities were apparently detected as long as the segment from −27 to +108 were contained (Fig. 1a). A similar result was obtained by a reporter assay using a mouse macrophage cell line, RAW264.7 (Fig. 1b). These results indicate that −27/−1 region of the promoter plays a pivotal role in the gene expression of *Ccl22* in both DCs and Macrophages. Then, we searched possible transcription factor-binding sites in this region using a database, JASPAR (http://jaspar.genereg.net/), and found that three Ets motifs: a typical Ets motif GGAA at −21/−18 (termed Site 1), and TTCTTCT containing two overlapping Ets-like motifs at −10/−4 (termed Site 2), are located within this region (Fig. 1c). To assess the necessity of these putative transcription factor-binding sites for promoter activity, we constructed mutant reporter vectors lacking Site1 and/or Site2. Although the introduction of a mutation at either Site 1 or Site 2 did not affect the luciferase activity, double mutation of both sites significantly reduced the activity (Fig. 1d). These results indicate that two sites containing Ets motifs, −21/−18 and −10/−4, are critical for transcriptional activity of the *Ccl22* promoter.

### PU.1 binding directly at the two proximal sites in the mouse *Ccl22* promoter

**Figure 2:**
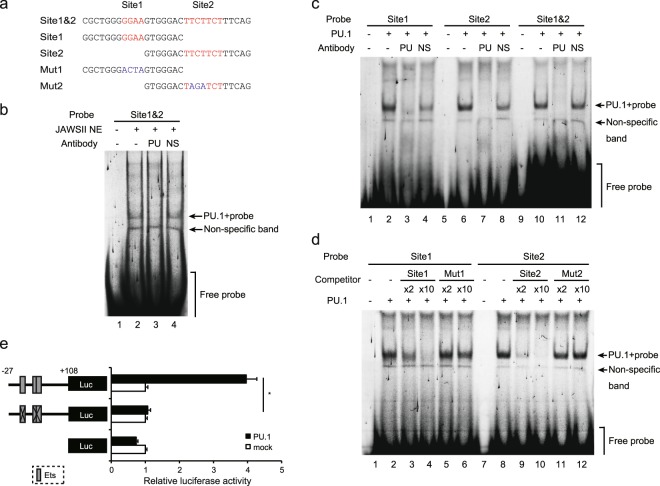
Identification of the PU.1 binding site. (**a**) Probes used in EMSAs. (**b**) The FLO-labeled Site 1&2 was incubated with the nuclear extracts prepared from JAWSII cells in the presence of either anti-PU.1 (PU) or non-specific (NS) Abs. (**c**) The FLO-labeled indicated probes were incubated with recombinant PU.1 protein in the presence of either anti-PU.1 (PU) or non-specific (NS) Abs. (**d**) The FLO-labeled indicated probes were incubated with recombinant PU.1 protein in the presence of 2-fold (×2) or 10-fold (×10) amounts of non-labeled identical WT or mutated competitor. After electrophoresis in 5% acrylamide gels, fluorescence was detected. (**e**) 293 T cells were transfected with either empty (mock) or PU.1-expression (PU.1) plasmids together with either WT or mutant reporter plasmids described in Fig. 1. At 48 h after transfection, luciferase and β-galactosidase activities were measured. Luciferase activities were normalized to β-galactosidase activities. Data are expressed as the ratio of the luciferase activity of the respective promoter-less plasmid-transfected cells. Results are shown as means + S.D.s (*n* = 3). Similar results were obtained in three independent experiments. *p < 0.05. Full-length gels with lower contrasts for (**b**–**d**), are included in Supplemental Information.

To identify transcription factor binding to the *cis*-enhancing elements containing Ets motifs (Site1 at −21/−18 and Site2 at −10/−4), EMSAs were performed using probes described in Fig. 2a. When a FLO-labeled probe containing two sites was mixed with the nuclear proteins extracted from JAWSII cells, several bands appeared on an electrophoretic gel (Fig. 2b lane 2). Among members of the Ets transcription factor family, PU.1/Spi1 especially plays critical roles in differentiation and cell type-specific gene expression of DCs and macrophages. Therefore, we hypothesized that PU.1 transactivates the *Ccl22* gene that is apparently expressed in monocytic lineages. To confirm that PU.1 binds to the probe, an anti-PU.1 Ab was added into the mixture of the probe and nuclear extracts. As shown in Fig. 2b lane 3, the most major band disappeared in the presence of an anti-PU.1 Ab, whereas the addition of a non-specific Ab did not affect this major band (*lane 4*). This result indicates that PU.1 dominantly binds to the *Ccl22* minimum promoter region in transcription factors expressed in monocytes. A clearer shift band containing PU.1 was detected in an EMSA, which was carried out by using a recombinant PU.1 protein generated using an *in vitro* transcription/translation system (Fig. 2c, *lanes 10*–*12*, and Supplemental Fig. 1). The specific band was also observed when FLO-labeled probes containing either Site 1 or Site 2 were used (Fig. 2c, *lanes 2*–*4 and 6*–*8*). We then performed an EMSA with competitors to identify the PU.1 binding sites in the probe sequence. The shifted band completely disappeared in the presence of an excess amount of the wild-type (WT) competitors, but not mutant competitors (Fig. 2d). These results suggest that PU.1 is capable of binding to −21/−18 GGAA and −10/−4 TTCTTCT sequences in the mouse *Ccl22* promoter. To further elucidate the involvement of PU.1 and the *cis*-elements in mouse *Ccl22* promoter activity, a luciferase assay was carried out using 293 T cells, because this non-hematopoietic cell line, in which PU.1 is not detected, is useful to evaluate the effect of exogenously expressed PU.1. Whereas the luciferase activity driven by the WT promoter was enhanced by co-expression of PU.1, introduction of mutations into both Site 1 and Site 2 completely diminished the transactivation effect of PU.1 on promoter activity (Fig. 2e). This result indicates that PU.1 transactivates the mouse *Ccl22* gene through binding to Site 1 and Site 2.

### PU.1/*Spi1* knockdown by siRNA transfection

**Figure 3:**
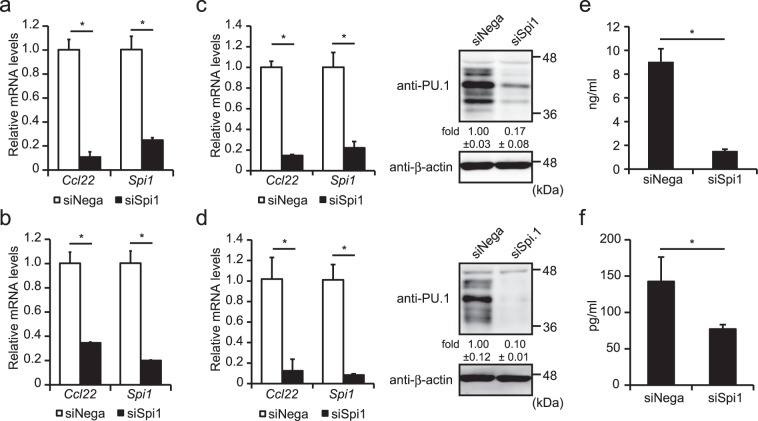
Effects of PU.1 knockdown on *Ccl22* expression in mouse DCs and macrophages. JAWSII (**a**), RAW264.7 (**b**), BMDCs (**c**,**e**), and BMDMs (**d** and **f**) were transfected with either negative control siRNA (siNega) or *Spi1* siRNA (siSpi1). At 48 h after transfection, relative mRNA levels were determined by quantitative RT-PCR after normalizing to mouse *Gapdh* mRNA levels. Data are expressed as the ratio of the expression level of the respective control siRNA-transfected cells. Western blotting analyses using anti-PU.1 Ab and anti-β-actin Ab were performed to evaluate the effect of *Spi1* siRNA on PU.1 protein levels in BMDCs (**c** right) and BMDMs (**d** right). (**e**,**f**) The concentration of the CCL22 protein produced from either siNega or siSpi1 transfected BMDCs (**e**) or BMDMs (**f**) was determined as described in the *Materials and Methods*. Results are shown as means + S.D.s (*n* = 3). Similar results were obtained in three independent experiments. *p < 0.05.

In order to determine the role of PU.1 in the expression of the *Ccl22* gene, we introduced *Spi1* siRNA into JAWSII and RAW264.7 cells. The introduction of *Spi1* siRNA markedly decreased mRNA levels of *Spi1* and *Ccl22* in JAWSII (Fig. 3a) and RAW264.7 (Fig. 3b). Further, we confirmed the effect of *Spi1* knockdown on *Ccl22* expression in primary mouse DCs and macrophages generated from bone-marrow cells by cultivation in the presence of GM-CSF and M-CSF, respectively. Under the PU.1-knocked-down condition (both of mRNA and protein levels), the *Ccl22* mRNA level was significantly decreased in BMDCs (Fig. 3c) and BMDMs (Fig. 3d). Furthermore, ELISA showed that the amount of CCL22 protein produced from BMDCs (Fig. 3e) and BMDMs (Fig. 3f) was markedly reduced by PU.1 knockdown. Although CCL22 production is enhanced during polarization to alternative activation macrophages (AAM), mRNA levels of other AAM makers, *Fizz-1* and *Ym-1* were not decreased but rather increased by *Spi1* knockdown (Supplemental Fig. 2), suggesting that reduction of *Ccl22* expression in *Spi1* knockdown cells is not simply due to a reduction of these cells from a less of an AAM phenotype. To evaluate the effect of Spi1 knockdown-mediated suppression of CCL22 production on the capacity to cause migration of Th2 cells, we performed a migration assay. We observed that the number of Th2 cells migrated to Spi1-knocked-down DCs was moderately lower than that to control DCs (Supplemental Fig. 3). We introduced other *Spi1* siRNAs into BMDCs to exclude the possibility of an off-target effect of *Spi1* siRNA and found that these two siRNAs also significantly suppressed the *Ccl22* mRNA level in parallel with the *Spi1* knockdown level (Supplemental Fig. 4). Taken together, these results suggest that PU.1 is involved in the gene expression of mouse *Ccl22* in DCs and macrophages.

### Binding of PU.1 to the mouse *Ccl22* promoter in DCs and macrophages

**Figure 4:**
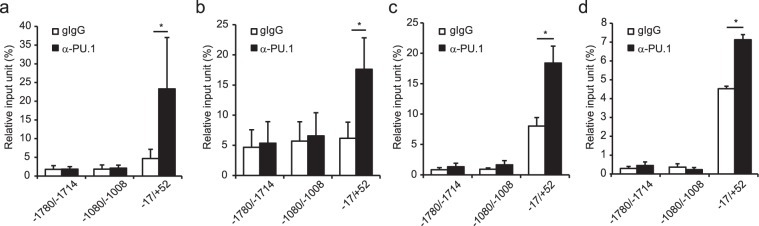
Analysis of the PU.1 binding region in the mouse *Ccl22* promoter. ChIP assay was performed using either goat IgG (gIgG) or anti-PU.1 Ab (PU.1) in BMDCs (**a**), BMDMs (**b**), splenic DCs (**c**), and peritoneal macrophages (**d**). The amounts of immunoprecipitated chromatin were determined by quantitative PCR amplifying the indicated region of the *Ccl22* promoter. Data are expressed as the percentage of the input for each ChIP assay. Results are shown as means + S.D.s (*n > 3*). Similar results were obtained in more than three independent experiments. *p < 0.05.

To investigate whether PU.1 binds to the *Ccl22* gene in CCL22-expressing cells, we performed ChIP assays using BMDCs (Fig. 4a) and BMDMs (Fig. 4b). A significant amount of PU.1 binding to −17/+52 was detected in DCs and macrophages, whereas PU.1 did not bind to further upstream regions, −1780/−1714 and −1080/−1008 (Fig. 4a,b). These results demonstrate the specific binding of PU.1 to the proximal region of the transcription start site of the mouse *Ccl22* gene.

Furthermore, we carried out ChIP assays using DCs and macrophages freshly isolated from mice. As expected, significant and specific binding of PU.1 to −17/+52 of the *Ccl22* promoter was detected in splenic DCs (Fig. 4c) and peritoneal macrophages (Fig. 4d). These results indicate that PU.1 trasactivates the *Ccl22* gene by binding to the proximal region of the promoter in DCs and macrophages *in vivo*.

### Involvement of IRFs in the gene expression of *Ccl22*

**Figure 5:**
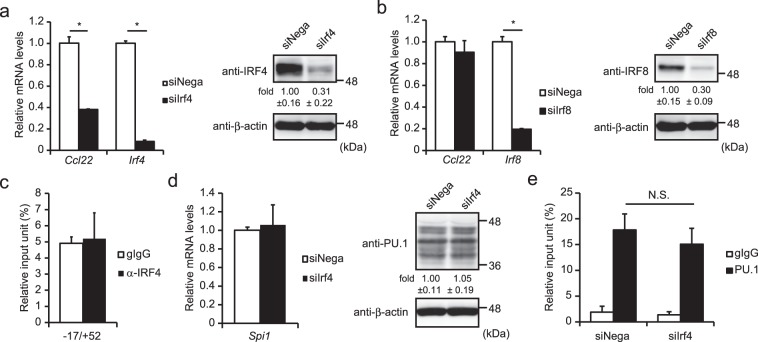
Involvement of IRFs in the expression of *Ccl22* in BMDCs. (**a**,**b**) BMDCs were transfected with negative control siRNA (siNega), Irf4 siRNA (siIrf4) (**a**), or Irf8 siRNA (siIrf8) (**b**). At 48 h after transfection, relative mRNA levels were determined by quantitative RT-PCR after normalizing to mouse *Gapdh* mRNA levels. Data are expressed as the ratio of the expression level of the respective control siRNA-transfected cells. Western blotting analyses were performed using transfectants, which were harvested at 48 h after transfection (right in **a**,**b**). (**c**) ChIP assay was performed by using either goat IgG (gIgG) or anti-IRF4 Ab (IRF4) in BMDCs. The amounts of immunoprecipitated chromatin were determined by quantitative PCR targeting the −17/+52 region of the *Ccl22* promoter. Data are expressed as the percentage of the input for each ChIP assay. (**d**) mRNA level (left) and protein level (right) of PU.1 in control (siNega) or *Irf4* knockdown cells (siIrf4). (**e**) PU.1 binding degree to −17/+52 in control (siNega) or *Irf4* knockdown cells (siIrf4) determined by ChIP assay. Results are shown as means + S.D.s (*n* = *3*). Similar results were obtained in three independent experiments. *p < 0.05.

In addition to the role as a monomeric transcription factor, PU.1 also regulates the target genes forming a heterodimer with IRF4 or IRF8. In order to investigate the involvement of IRFs in the transcriptional regulation of *Ccl22*, we introduced siRNA against either *Irf4* or *Irf8* into BMDCs. Quantitative PCR showed that *Irf4* knockdown significantly decreased the mRNA level of *Ccl22* under the condition, in which levels of *Irf4* mRNA and IRF4 protein were reduced (Fig. 5a). In contrast, *Irf8* siRNA did not affect the *Ccl22* mRNA level even though levels of *Irf8* mRNA and IRF8 protein were decreased (Fig. 5b). To examine whether IRF4 co-localizes to the minimum promoter region of the *Ccl22* gene with PU.1, we performed a ChIP assay using an anti-IRF4 Ab and quantified the amount of the immunoprecipitated chromosomal DNA by amplifying the region −17/+52. However, we did not detect significant binding of IRF4 around the identified PU.1 binding sites (Fig. 5c). We evaluated the effect of *Irf4* siRNA on the expression and/or binding of PU.1 to −17/+52. As shown in Fig. 5d, levels of mRNA and protein of PU.1 were not affected by *Irf4* siRNA. The amount of PU.1 binding to the region −17/+52 was not significantly reduced by *Irf4* siRNA (Fig. 5e). These observations suggest that the involvement of IRF4 in the gene expression of *Ccl22* is not due to the formation of a transcription complex with PU.1 around the transcriptional start site. IRF4 may transactivate the *Ccl22* gene via another region on this gene or may indirectly regulate *Ccl22* gene expression through transactivation of another transcription factor(s).

### Effect of TLR-mediated stimulation on the recruitment of PU.1 to the gene

**Figure 6:**
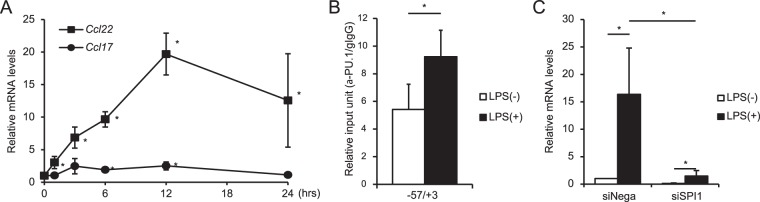
Involvement of PU.1 in the expression of *Ccl22* in mature BMDCs. (**a**) BMDCs were stimulated with 1 μg/ml LPS for the indicated period. Relative *Ccl22* and *Ccl17* mRNA levels were determined by quantitative RT-PCR after normalizing to mouse *Gapdh* mRNA levels. Data are expressed as the ratio of the expression level of the non-stimulated cells. (**b**) ChIP assay was performed using either goat IgG (gIgG) or anti-PU.1 Ab (α-PU.1) in BMDCs stimulated with 1 μg/ml LPS for 24 hours. The amounts of immunoprecipitated chromatin were determined by quantitative PCR targeting the −17/+52 region of the *Ccl22* promoter. Data are expressed as the ratio of α-PU.1 to gIgG (α-PU.1/gIgG). (**c**) BMDCs were transfected with either negative control siRNA (siNega) or *Spi1* siRNA (siSpi1). At 24 hours after transfection, cells were stimulated with 1 μg/ml LPS and further incubated for 24 hours. Relative mRNA levels were determined by quantitative RT-PCR after normalizing to mouse *Gapdh* mRNA levels. Data are expressed as the ratio of the expression level of the control siRNA-transfected immature BMDCs. Results are shown as means + S.D.s (*n* > *3*). Similar results were obtained in more than three independent experiments.

DCs and macrophages dramatically alter their gene expression in response to stimulation signaling such as TLR ligands. As shown in Fig. 6a, the mRNA level of *Ccl22* was markedly increased in response to LPS stimulation, in contrast to a slight increase in the *Ccl17* mRNA level. Thus, we performed a ChIP assay to investigate whether the LPS-induced upregulation of *Ccl22* transcription reflects the PU.1 binding ability to the *Ccl22* promoter in DCs. As shown in Fig. 6b, the PU.1 binding level at the region −17/+52 in mature BMDCs was approximately 1.8-fold of that of immature BMDCs. We also confirmed that the LPS-induced increase of the *Ccl22* mRNA level was significantly attenuated by siRNA-mediated *Spi1* knockdown (Fig. 6c). These results demonstrate that PU.1 recruitment to the *Ccl22* promoter is augmented by LPS-dependent maturation, thereby increasing the transcription of *Ccl22*.

### Involvement of PU.1 in expression of the human *CCL22* gene

**Figure 7:**
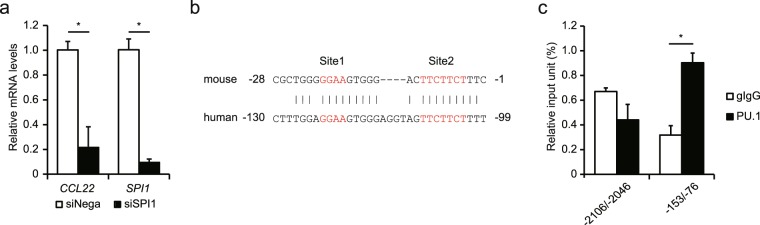
Involvement of PU.1 in the expression of the human *CCL22* gene. (**a**) mRNA levels of *CCL22* and *SPI1* in *SPI1* siRNA (siSPI1) or control siRNA (siNega) introduced THP-1 cells. (**b**) Alignment of nucleotide sequences of human and mouse *CCL22* genes. (**c**) ChIP assay was performed using either control IgG (gIgG) or anti-PU.1 Ab (α-PU.1) in THP-1 cells. The amounts of immunoprecipitated chromatin were determined by quantitative PCR targeting the −153/−76 (including *cis*-elements) or −2106/−2046 (*cis*-control) of the *CCL22* promoter. Results are shown as means + S.D.s of three independent experiments.

To investigate whether the role of PU.1 in *Ccl22* expression in mouse monocytes is translatable to human biology, we evaluated the effect of *SPI1* siRNA on *CCL22* expression level in human monocyte cells, THP-1. As shown in Fig. 7a, *SPI1* siRNA effectively suppressed *SPI1* mRNA level and subsequently reduced *CCL22* mRNA level. When nucleotide sequence of the human *CCL22* gene was compared with that of mouse, we found that the elements similar to Site1 and Site2 exist on the human *CCL22* promoter (Fig. 7b). By ChIP assay, we confirmed that PU.1 bound to this region in human monocyte cells (Fig. 7c). These results suggest that PU.1 is a common transactivator for the *CCL22 (Ccl22)* gene in human and mouse.

## Discussion

The Ets family transcription factor PU.1 is essential for myeloid cell development. Several studies have demonstrated that PU.1 plays critical roles in DC and macrophage functions^13,14,16,20^. In the current study, we provided evidence that PU.1 is involved in *Ccl22* expression in DCs and macrophages.

Using a reporter assay, we demonstrated that transcriptional regulatory elements of the *Ccl22* gene located within −27/+108 and two putative PU.1 binding elements were critical for transcriptional activity. *Ccl22* expression was markedly decreased by *Spi1* knockdown in all investigated cell types. ChIP assays showed that PU.1 bound to the proximal region of the *Ccl22* promoter. From these results, we demonstrate that PU.1 is one of the most important transcription factors in the gene expression of *Ccl22*. Although transcriptional activity was significantly suppressed by mutation of the two PU.1 binding motifs in the luciferase assay, the activity was still higher than that of those without the promoter. This result indicates that another transcription factor(s) is involved in the gene expression of *Ccl22* by binding within the −27/+108 region. Indeed, CCL22 is also expressed in non-hematopoietic cells, such as keratinocytes, which do not express PU.1. Previous studies have demonstrated that NF-κB and STAT1 are involved in the IFN-γ/TNF-α-dependent upregulation of *CCL22* in human keratinocytes^21,22^. Considering that our reporter assay was carried out in a steady state, these molecules were not likely to enhance the transcriptional activity via binding to the −27/+108 region. It will be intriguing to further investigating which transcription factors regulate *Ccl22* gene expression.

Even though ChIP assays were carried out using JAWSII and BMDCs without inducing maturation, we detected significant amount of PU.1 binding to the *Ccl22* promoter. These results suggest that PU.1 plays a role as an activator of basal transcription of the *Ccl22* gene in DCs, which produce a large amount of CCL22 even in the absence of inflammatory signaling. We also exhibited that PU.1 binding levels at the identified region were increased in response to LPS stimulation and subsequent upregulation of *Ccl22* was significantly attenuated by *Spi1* knockdown. These results suggest that PU.1 also plays an important role in the transactivation of *Ccl22* upon DC maturation.

Here we used BMDMs and peritoneal macrophages as macrophage models. Macrophages are classified into two groups, M1 macrophages and M2 macrophages, depending on their expression of surface makers, cytokines, and chemokines. *Ccl22* is one of the M2 macrophage-markers, and BMDMs generated with M-CSF are thought to exhibit an M2-like phenotype^23^. Indeed, we demonstrated that PU.1 is involved in the transcriptional regulation of *Ccl22* by binding to the promoter in BMDMs. Recent studies have demonstrated that characteristic transcription factors like Spi-C and GATA6 are essential for differentiation of splenic and peritoneal macrophages, respectively^24,25^, suggesting that tissue-resident macrophages are functionally distinct. Although we showed that PU.1 marginally bound to the *Ccl22* promoter in peritoneal macrophages, it is possible that PU.1 binding level is altered depending on where macrophages reside. Further analyses are required to elucidate this.

Previously, Chang *et al*. demonstrated that PU.1 promotes the development of Th9^26^ and subpopulation of Th2^27^ that express CCL22. PU.1 has also been described in promoting alternative activation of macrophages that produce CCL22^28^. In the current study, it was revealed that the *CCL22* (*Ccl22*) gene, which has been reported to be under the control of PU.1, is directly transactivated by PU.1 via the *cis*-elements. Although Th cells were not examined in our study, the *CCL22* gene may be a direct target of PU.1 in Th cells.

Previous studies have demonstrated that serum levels of CCL22 and CCL17 are significantly elevated and correlated with disease severity in patients with Atopic dermatitis^2,29–31^. Moreover, administration of CCR4 blocking antibody to the airway inflammation model abolished asthmatic symptoms^32^. According to these studies, targeting the interactions between CCL17/CCL22 and CCR4 may be a useful approach for controlling allergic diseases. We previously reported that injection of *Spi1* siRNA significantly suppressed contact hypersensitivity of mice^13^. Considering that Th2-related genes such as *Tnsf4* and *Ccl22* are also transactivated by PU.1 in DCs, *Spi1* knockdown may be a favorable strategy for allergic diseases.

## Materials and Methods

### Mice and cells

Bone marrow-derived DCs (BMDCs) and macrophages (BMDMs) were generated from BALB/c mice (Japan SLC, Hamamatsu, Japan) as previously described^16,33,34^.

Mouse DC line JAWSII, mouse macrophage line RAW264.7, and human monocyte cell line THP-1 were maintained as previously described^13,16,35^. All animal experiments were performed in accordance with the approved guidelines of the Institutional Review Board of Tokyo University of Science, Tokyo, Japan. The Animal Care and Use Committees of Tokyo University of Science specifically approved this study.

### Isolation of splenic DCs and peritoneal macrophages

CD11c microbeads and an auto MACS Pro separator (Miltenyi Biotech, Tubingen, Germany) were used to isolate DCs from mouse spleen. Peritoneal macrophages were obtained as adhesive cells by incubating mouse peritoneal cells with 10% FCS-DMEM for 1 hour.

### Introduction of siRNA into cells

*Spi1* siRNA (Stealth Select RNAi, Sfpi1-MSS247676), *Irf4* siRNA (MSS205499), *Irf8* siRNA (MSS236848), *SPI1* siRNA (SFPI1-HSS186060) and control siRNA purchased from Invitrogen (Carlsbad, CA) were introduced into BMDCs or BMDMs as previously described^16^.

### Quantitative RT-PCR

Quantitative RT-PCR was performed as previously described^36^. The TaqMan primers are listed as follows: *Spi1*, Mm00488142_m1; *Irf4*, Mm00516431_m1; *Irf8*, Mm00492567_m1; *Gapdh*, 4352339E; *SPI1*, Hs02786711_m1; *GAPDH*, 4326317E. The nucleotide sequences of primers used for detection of the mouse *Ccl22* and human *CCL22* mRNAs are as follows: mCcl22-forward; 5′-AAGCCTGGCGTTGTTTTGAT-3′, mCcl22-reverse; 5′-CCTGGGATCGGCACAGATA-3′, hCCL22-forward; 5′-CCCTACGGCGCCAACAT-3′, and hCCL22-reverse; 5′-CAGACGGTAACGGACGTAATCA-3′.

### Western blotting

Western blotting analysis was performed as previously described^20^. Following antibodies were used: anti-PU.1 antibody (D-19), anti-IRF4 antibody (M-17), and anti-IRF8 antibody (C-19), (Santa Cruz Biotechnology, Santa Cruz, CA), and anti-β-actin antibody (AC-15, Sigma-Aldrich).

### ELISA

The concentration of mouse CCL22 protein was determined using an ELISA kit (MCC220, R&D systems, Minneapolis, MN).

### Luciferase assay

A series of reporter plasmids carrying the wild type or mutant mouse *Ccl22* promoters were generated as previously described^16^ using primer sets listed in Supplemental Table 1.

Transfections of JAWSII, RAW264.7, and 293 T cells were performed as previously described^16^. After 48 hours, luciferase activity was determined using ARVO Light (Perkin Elmer, Waltham, MA) and a Dual-luciferase assay kit (Promega), as previously described^37^.

### EMSA

Nuclear proteins were prepared from JAWSII cells. PU.1 protein was synthesized using a TNT T7 quick-coupled transcription/translation system (Promega) and pCR-PU.1 as a template. After incubation of the proteins and fluorescein-labeled oligonucleotides, electrophoretic mobility shift assay was performed as previously described^16,38^.

### ChIP assay

ChIP assays were performed as previously described^37^. Antibodies against PU.1 and IRF4 were the same as those used in Western blotting analysis. Goat IgG (#02-6202, Invitrogen) was used as control antibody. The amount of immunoprecipitated or input DNA was quantified by a real-time PCR system using the primer sets (see Supplementary Table 2).

### Statistical analysis

Statistical analysis was performed as previously described^16^.

## Supplementary information

Supplemental information
