# Supplementary material for: A transcription factor PU.1 is critical for Ccl22 gene expression in dendritic cells and macrophages
Source: Sci Rep. 2019 Feb 4;9:1161. doi: 10.1038/s41598-018-37894-9 (PMC6361964; doi:10.1038/s41598-018-37894-9)
Supplement: Supplementary file 1 — Supplemental information [file 41598_2018_37894_MOESM1_ESM.pdf]

A transcription factor PU.1 is critical for *Ccl22* gene expression in dendritic cells and macrophages

Takuya Yashiro, Shiori Nakano, Kurumi Nomura, Yuna Uchida, Kazumi Kasakura, and Chiharu Nishiyama

Department of Biological Science and Technology,

Faculty of Industrial Science and Technology, Tokyo University of Science,

6-3-1 Niijuku, Katsushika-ku, Tokyo, 125-8585, Japan

Supplementary Table 1. Sequence of primers used in construction of reporter vectors.

5' position	Direction	Sequence
-597	Forward	CATAggtaccGCACACTCACTCTGGCTCGC
-245	Forward	CGTGggtaccATCCATGTGCCAGTGGCCAC
-101	Forward	CCACggtaccACAATCCTCCGGAGACCCAC
-65	Forward	CGTAggtaccAGTCCCCTCTGAGTAGTTC
-27	Forward	CGTAgattccGCTGGGGGAAGTGGGACT
+108	Reverse	GTGCagatctGTCAGGTGTCTAATGACCAG

Mutation site	Direction	Sequence
Site1	Forward	CTGGCCGGTACCGCTGGGACTAGTGGGACTTCTTCTTTCAG
	Reverse	CTGAAAGAAGAAGTCCCACTAGTCCCAGCGGTACCGGCCAG
Site2	Forward	CCGCTGGGGGAAGTGGGACTAGATCTTTCAGGACATGAATGTC
	Reverse	GACATTCATGTCCTGAAAGATCTAGTCCCACTTCCCCCAGCGG
Site1&2	Forward	CCGCTGGGACTAGTGGGACTAGATCTTTCAGGACATGAATGTC
	Reverse	GACATTCATGTCCTGAAAGATCTAGTCCCACTAGTCCCAGCGG

Supplementary Table 2. Sequence of primers used in ChIP assay.

<Mouse>

Amplified region	Direction	Sequence
-1780/-1714	Forward	CCAAAGGGTTTTGAGCTCTCA
	Reverse	GCTACTTCAGAGGGTAAAAAAAAAGG
-1080/-1008	Forward	AAAGCCCACCGCATCATG
	Reverse	GCTGACTGTGTGCTCCAGGTT
-17/+52	Forward	GTGGGACTTCTTCTTTCAGGACAT
	Reverse	CCCACTAGTTCAGGCACCTCTT

<Human>

Amplified region	Direction	Sequence
-2106/-2046	Forward	ACCAGCCCTGGCAACATAGT
	Reverse	CGCCCTGCTCATTTTTATTTCT
-153/-76	Forward	CCCCGCCAAAGAGAATTTC
	Reverse	GAGTCACTTGACATTCACATCTCAAA

*+1; Transcription start site

Supplemental figure

Materials and Methods

Materials

For detection of the mouse *Fizz-1/Retnla* and *Ym-1/Chil3* mRNAs, the following primers were used: mFizz1-F, 5’-ATCCCATGGCGTATAAAAGCAT-3’,

mFizz1-R, 5’-GGGCAGAATCTCAGGAAAGGTT-3’,

mYm1-F, 5’-TCTGGTGAAGGAAATGCGTAAA-3’,

mYm1-R, 5’-GCAGCCTTGGAATGTCTTTCTC-3’.

Spi1/Pu.1 siRNA #1, 2 (Stealth Select RNAi, MSS247678 and MSS277025, respectively), and negative control siRNA (Stealth Negative Control Hi GC, 12935-400) were obtained from Invitrogen (Carlsbad, CA).

Methods

Preparation of Th2 cells and Migration assay

Naïve CD4$^+$ T cells prepared from spleen using a MojoSort Ms CD4 Naïve T cell isolation kit (#480040, BioLegend) were polarized to Th2 cells by cultivation in the medium supplemented with 20 ng/ml recombinant mouse IL-4 (#094-03944, Wako) and 10 μg/ml anti-IL-12p40 antibody (C17.8, BioLegend) in dishes coated with 1 μg/ml anti-CD3 antibody (145-2C11, TONBO) and 10 μg/ml anti-CD28 antibody (37.51, TONBO) for 5 days. The generated Th2 cells (5 x 10^5/100 μl) were subjected on upper site of a transwell (5 μm pore size #3421, Corning), which were placed on a well filled

with 500 μl culture supernatant of siRNA-introduced BMDCs. After 3 h incubation, the cell number on lower site of a transwell was counted.

Figure legends

Sup. Fig. 1. Western blotting profile of in vitro translated protein

Five μl of 20-fold diluted *in vitro* translated reaction mixture was loaded and detected by anti-PU.1 antibody.

Sup. Fig. 2. Expression of Fizz-1 (*Retnla*) and Ym-1 (*Chil3*) in *Spi1* siRNA introduced BMDMs

BMDMs were transfected with either negative control siRNA (siNega) or *Spi1* siRNA (siSpi1). At 48 h after transfection, relative mRNA levels were determined by quantitative RT-PCR after normalizing to mouse *Gapdh* mRNA levels. Data are expressed as the ratio of the expression level of the respective control siRNA-transfected cells. Results are means + S.D.s ($n=3$). Similar results were obtained in three independent experiments. *, $p < 0.05$.

Sup. Fig. 3. Effect of *Spi1* siRNA on the migration activity

In vitro differentiated Th2 cells were seeded in the upper chambers of the transwell. The lower chambers were filled with culture supernatants from BMDCs transfected with either negative control siRNA (siNega) or *Spi1* siRNA (siSpi1) or culture medium as negative control (N.C.). After 3 h incubation, the cell number on lower site was

counted.

The numbers of migrated T cells are shown.

Sup. Fig. 4. Effect of *Spi1* siRNA on the expression of *Ccl22*

BMDCs were transfected with either negative control siRNA (siNega) or *Spi1* siRNA (siSpi1). At 48 h after transfection, relative mRNA levels were determined by quantitative RT-PCR after normalizing to mouse *Gapdh* mRNA levels. Data are expressed as the ratio of the expression level of the respective control siRNA-transfected cells. Results are means + S.D.s ($n=3$). Similar results were obtained in three independent experiments. *, $p < 0.05$.

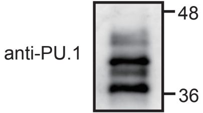

Sup. Fig. 1

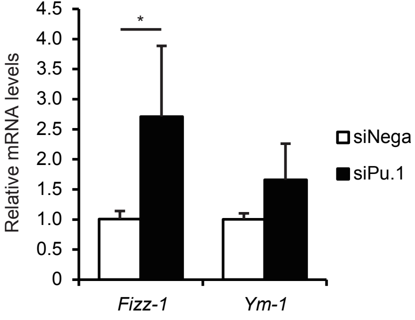

4.5
4.0
3.5
3.0
2.5
2.0
1.5
1.0
0.5
0
Relative mRNA levels
*
siNega
siPu.1
Fizz-1
Ym-1

Sup. Fig. 2

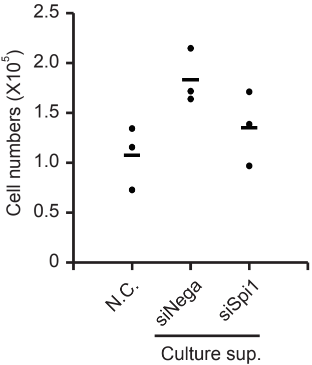

2.5
2.0
1.5
1.0
0.5
0
Cell numbers (X10^5)
N.C.
siNega
siSpi1
Culture sup.

Sup. Fig. 3

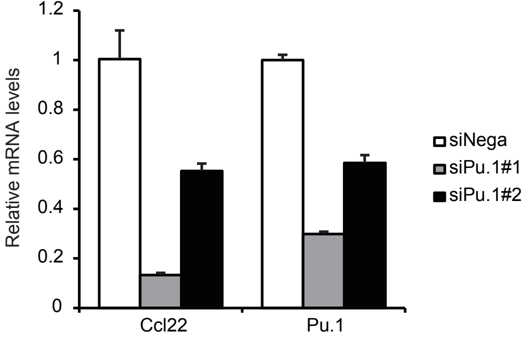
1.2
1
0.8
0.6
0.4
0.2
0
Relative mRNA levels
Ccl22
Pu.1
siNega
siPu.1#1
siPu.1#2

Sup. Fig. 4

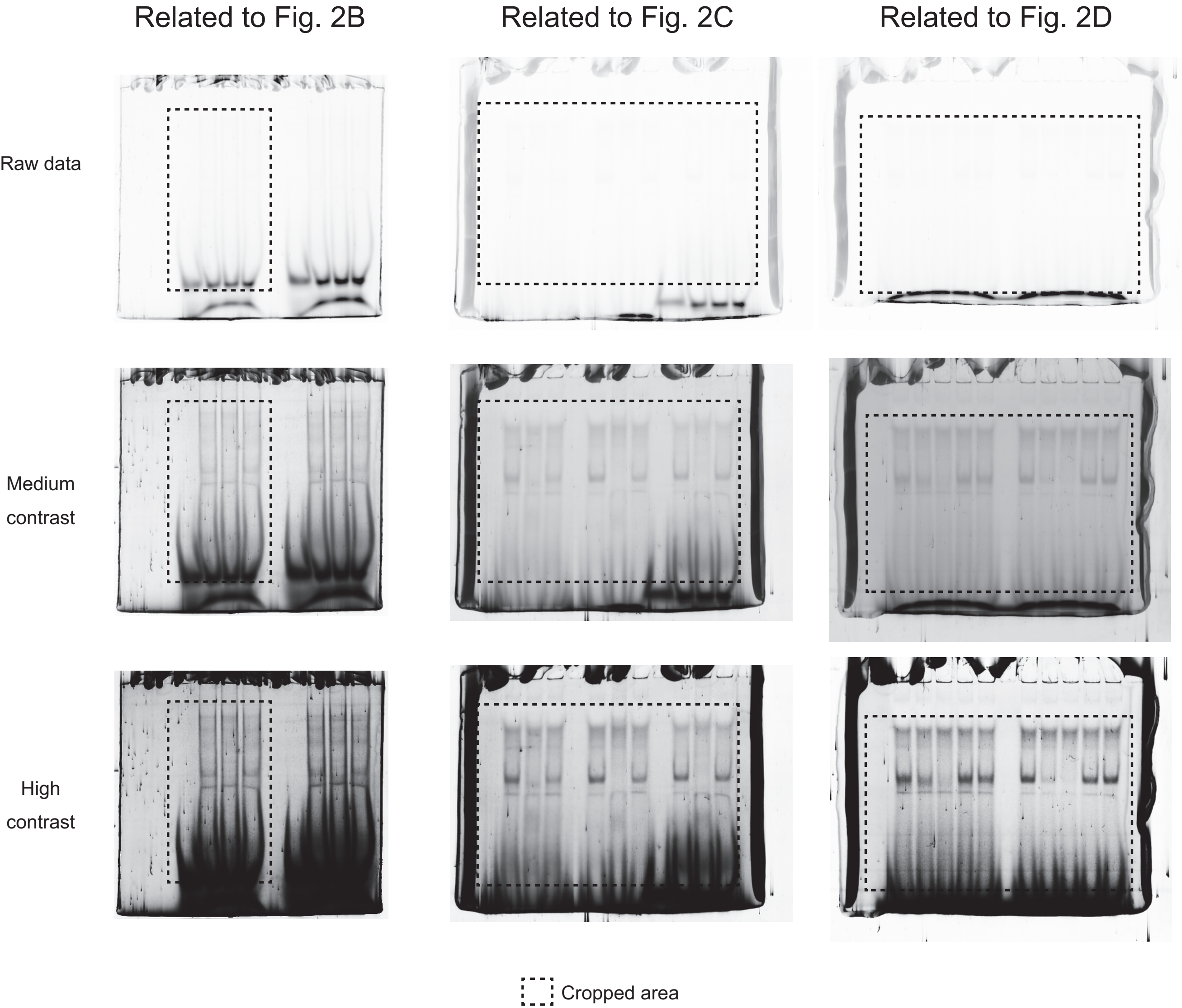
Related to Fig. 2B
Related to Fig. 2C
Related to Fig. 2D
Raw data
Medium contrast
High contrast
Cropped area

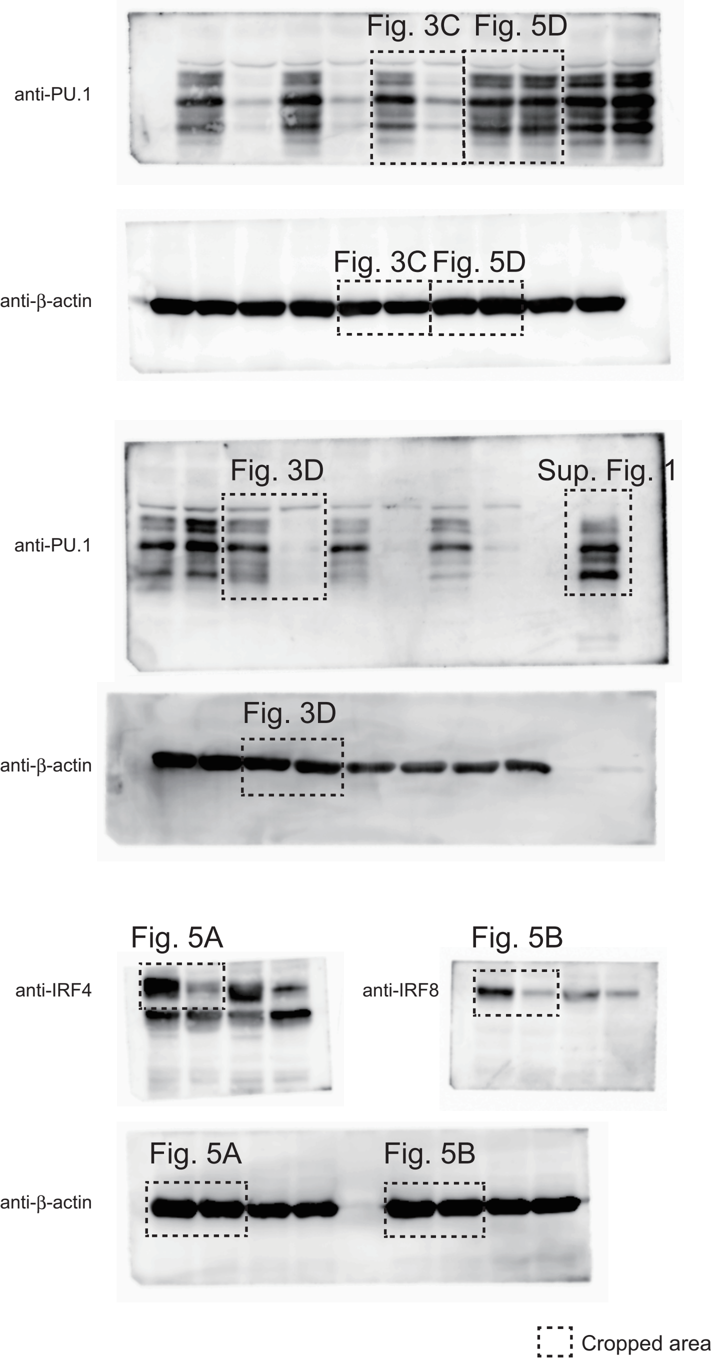
anti-PU.1
Fig. 3C
Fig. 5D
anti-β-actin
Fig. 3C
Fig. 5D
anti-PU.1
Fig. 3D
Sup. Fig. 1
anti-β-actin
Fig. 3D
Fig. 5A
anti-IRF4
Fig. 5B
anti-IRF8
anti-β-actin
Fig. 5A
Fig. 5B
Cropped area
